# GLOBAL RESEARCH TRENDS IN AQUATIC EXERCISE THERAPY FOR MUSCULOSKELETAL DISORDERS: A BIBLIOMETRIC ANALYSIS

**DOI:** 10.2340/jrm.v57.42473

**Published:** 2025-04-29

**Authors:** Wei GUO, Xiaowei FENG, Weiping DU

**Affiliations:** 1 School of Physical Education, Ningxia Normal University, Guyuan; 2 Center for Sports and Health Research, Ningxia Normal University, Guyuan; 3 School of Physical Education/School of Football, Hainan Normal University, Hainan, China

**Keywords:** aquatic exercise therapy, musculoskeletal disorders, bibliometric analysis, publication trends, thematic evolution, rehabilitation, quality of life, osteoarthritis, rheumatoid arthritis

## Abstract

**Objective:**

Aquatic exercise therapy has gained recognition as a valuable non-pharmacological intervention for managing musculoskeletal disorders. Despite the growing body of evidence supporting its efficacy, research on aquatic exercise therapy remains fragmented, with limited understanding of key trends, influential studies, and evolving themes within the field. This study aims to conduct a comprehensive bibliometric analysis to identify publication trends, key authors, and the evolution of research themes in aquatic exercise therapy for musculoskeletal disorders.

**Methods:**

A total of 117 articles were selected based on predefined search terms and inclusion criteria, resulting in 3,985 citations, with an average of 35.9 citations per article and an H-index of 37.

**Results:**

Publications surged between 2013 and 2024, accounting for 88.3% of total output. Peaks occurred in 2019 (*n* = 14) and 2022 (*n* = 368 citations). Key contributors include Mariana Arias Avila and Basia Belza, with journals such as *BMC Musculoskeletal Disorders* and *Arthritis & Rheumatism-Arthritis Care & Research* playing pivotal roles. Research trends shifted from disease-specific studies to broader quality-of-life outcomes, with keywords such as “rheumatoid arthritis“, “balneotherapy“, and “quality of life” emerging as focal points.

**Conclusion:**

The findings underscore the growing importance of aquatic exercise therapy in clinical rehabilitation and suggest that future research should focus on long-term outcomes, underrepresented populations, and the integration of aquatic exercise therapy with emerging rehabilitation technologies.

## LAY ABSTRACT

With the ageing of the world population, musculoskeletal diseases affecting muscles, joints, and bones are becoming more and more common. Many people have begun to use non-drug therapy, such as aquatic exercise therapy, to treat these diseases. This study analyses the trends and key areas of aquatic exercise therapy research in musculoskeletal diseases, using 117 studies published from 1972 to 2024. Our analysis shows that since 2013 people’s interest in aquatic exercise therapy has increased significantly, which may be due to the increasing demand for effective and non-drug therapies for the elderly. Early research focused on specific diseases such as rheumatoid arthritis, but recent research has emphasized improving the overall quality of life and standardizing treatment methods to make aquatic exercise therapy more extensive and effective. These findings provide important insights for developing better and more accessible aquatic exercise therapy programmes to help musculoskeletal diseases patients stay active and improve their quality of life.

Musculoskeletal disorders (MSDs) are a group of conditions that affect the muscles, bones, joints, and connective tissues, leading to pain, reduced mobility, and decreased quality of life (1–4). These disorders are among the most common causes of disability worldwide, affecting millions of people each year. The global prevalence of MSDs is increasing, driven by factors such as ageing populations, sedentary lifestyles, and an increase in chronic conditions such as obesity and diabetes (5). According to the World Health Organization (WHO), MSDs account for a significant portion of the global burden of disease, with conditions like osteoarthritis, low back pain, and rheumatoid arthritis ranking among the top contributors to disability-adjusted life years (6).

The management of MSDs is complex and often requires a multidisciplinary approach, combining pharmacological treatments, physical therapy, and, in some cases, surgical interventions (7). Traditionally, non-pharmacological treatments, including physical activity and exercise, have played a central role in MSD management (8). These interventions aim to reduce pain, improve function, and enhance overall well-being. However, the effectiveness of these treatments varies, and patients often require individualized care to address the specific challenges posed by their condition.

Aquatic exercise therapy (AET) has emerged as a promising non-drug intervention for the rehabilitation of patients with MSDs. AET involves exercise performed in water, which provides a unique therapeutic environment that can reduce the load on joints, improve circulation, enhance muscle strength, and improve flexibility, all while minimizing the risk of injury (9). Water’s buoyancy reduces the stress on the musculoskeletal system, making AET particularly effective for individuals with osteoarthritis, fibromyalgia, back pain, and other chronic musculoskeletal conditions that may be aggravated by traditional land-based exercise (10).

Over the past few decades, AET has gained increasing attention in the field of rehabilitation as an alternative or complementary therapy for managing MSDs. Several studies have demonstrated its effectiveness in reducing pain, improving physical function, and enhancing overall quality of life in patients with various musculoskeletal conditions (11, 12). The therapeutic properties of water, including hydrostatic pressure, buoyancy, and resistance, make it an ideal medium for exercise, especially for individuals with limited mobility or those recovering from surgery or injury. Moreover, AET offers psychological benefits by fostering a sense of well-being, reducing anxiety, and promoting social interaction, all of which are essential components of a holistic rehabilitation approach (13). Interventions for MSDs are mainly conservative measures, including drug intervention and non-drug intervention. In the traditional conservative treatment of MSDs, drugs, injections, or electroshock therapy are often expensive (14), and the effect is not good (15). As one of the non-drug interventions for MSDs, the unique characteristics of aquatic exercise may enable people to undertake exercise that they cannot do on land. The benefits provided by the aquatic environment are mainly related to the physical characteristics of water, such as density, hydrostatic pressure, and buoyancy (16), which can provide such benefits as increasing muscle strength (17, 18) and reducing the stress on bones, joints, and muscles (19). Studies have shown that AET is beneficial to Parkinson’s disease (20), multiple sclerosis (21), chronic kidney disease (22), and so on. In addition, this method can also significantly improve the pain management, functional activity, and overall quality of life of patients with MSDs, such as chronic low back pain (23), ankylosing spondylitis (24), and knee osteoarthritis (25). Therefore, as a new physical therapy method, AET is increasingly welcomed by doctors and patients with MSDs, and the publication of related scientific research is also increasing yearly.

At present, there have been related systematic reviews and meta-analyses to sort and summarize the studies on AET of MSDs (26–28). Nevertheless, these studies mostly focus on evaluating specific diseases or individual curative effects and fail to systematically reveal the development trend and research hotspots of AET research from a macro perspective. Bibliometric analyses allow for the identification of key trends, influential authors, major journals, and thematic shifts in the literature, offering valuable insights into the development of the field and guiding future research (29). However, there is still a lack of relevant bibliometrics research on applying AET in MSDs. To make up for this gap, this study aims to comprehensively evaluate the research status of AET in treating MSDs through bibliometric analysis and reveal the global research hotspots and evolution trends in this field.

This study aims to fill this gap by performing a bibliometric analysis of the literature on aquatic exercise therapy for musculoskeletal disorders, with a focus on identifying key publication trends, the distribution of research across major authors and journals, and the evolution of research themes over time. Specifically, this analysis will explore the growth of AET research from 1972 to 2024, examine the geographic distribution of research contributions, and identify the most cited articles in the field. Additionally, this study will highlight the changing research priorities, from disease-specific interventions to broader outcomes related to functional rehabilitation and quality of life, providing valuable information for researchers and clinicians working to optimize AET for MSDs. By addressing these objectives, the study aims to contribute to a better understanding of the current state of AET research for MSDs, identify key gaps in the literature, and provide recommendations for future research directions. This bibliometric analysis will also serve as a valuable resource for researchers, healthcare providers, and policymakers involved in the development and implementation of AET programmes for the management of musculoskeletal disorders.

## Methods

### Data collection and search strategies

This study conducted a bibliometric analysis using data sourced from the Web of Science Core Collection (WoSCC), a widely recognized database that offers comprehensive coverage of high-impact scientific publications across various disciplines. As no human or animal subjects were involved, ethical approval was not required. The study aimed to provide a comprehensive overview of global research trends in AET for MSDs by analysing publication trends, author contributions, journal impact, and thematic evolution over the past 5 decades.

The literature search was conducted on 18 February 2025, following a well-defined search strategy using Boolean operators “AND” and “OR” to ensure a balance between specificity and comprehensiveness in retrieving relevant studies. The search query was designed to capture publications related to AET and MSDs by focusing on titles containing key terms associated with aquatic exercise interventions and musculoskeletal conditions. The initial search query was formulated as follows:

Search query: TI = (hydrotherapy OR aquatic exercise OR water-based exercise) AND (musculoskeletal disorders OR back pain OR neck pain OR arthritis OR osteoarthritis OR joint pain OR fracture OR fibromyalgia OR rheumatoid arthritis OR shoulder pain).

The search terms were selected based on a thorough review of the existing literature, consultation with subject matter experts, and consideration of terminological variations found in prior studies. Keywords such as “hydrotherapy” and “headache” were excluded from the revised search strategy to ensure that the retrieved records focused specifically on exercise-based interventions rather than passive water therapies or conditions not directly related to the musculoskeletal system. The rationale for keyword selection was to include a diverse but relevant range of musculoskeletal conditions that are commonly managed with aquatic exercise therapy while ensuring that the results remained within the scope of the study.

To ensure the relevance and quality of the dataset, the following inclusion and exclusion criteria were applied. Inclusion criteria: (*i*) publications published between 1972 and 2024, covering the evolution of AET in MSDs; (*ii*) articles published in peer-reviewed journals indexed in WoSCC; (*iii*) literature categorized as original research articles and systematic reviews relevant to the topic; (*iv*) articles published in English, to maintain consistency and avoid translation biases. Exclusion criteria: (*v*) non-English articles (*n* = 5); (2) articles published in 2024, due to incomplete indexing at the time of search; (3) conference abstracts, editorials, and opinion papers.

**Fig. 1:**
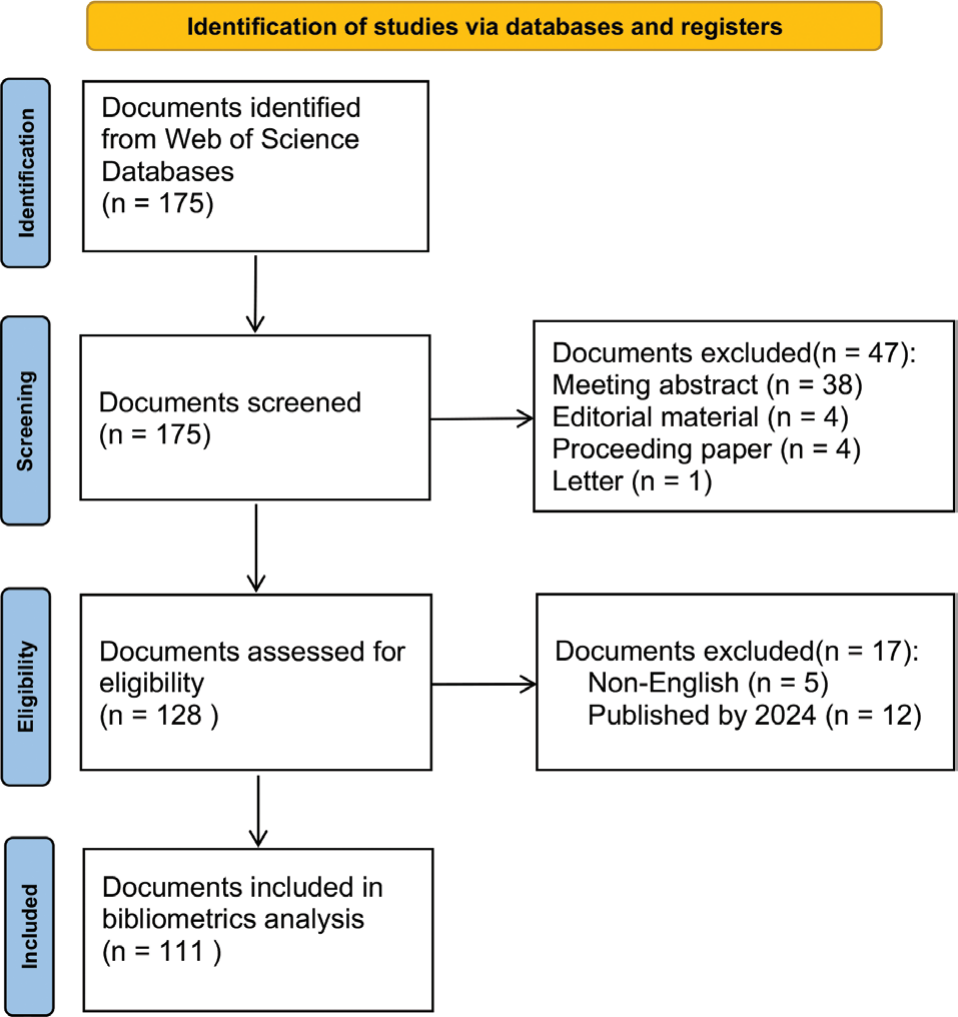
PRISMA flowchart.

After retrieving an initial total of 175 records from the WoSCC database, a rigorous screening process was implemented in accordance with the Preferred Reporting Items for Systematic Reviews and Meta-Analyses (PRISMA) guidelines (30). The screening process involved the exclusion of publications from the year 2024 due to incomplete data indexing at the time of the search, as well as the removal of non-English articles (*n* = 5) to maintain consistency and reduce language bias. To ensure the quality and relevance of the dataset, only original research articles (*n* = 95) and systematic reviews (*n* = 16) were included, resulting in a final sample of 111 articles that were subjected to detailed analysis. The selection process is visually summarized in the PRISMA flowchart (Fig. 1), illustrating the step-by-step exclusion and inclusion criteria applied during data curation.

### Data analysis strategy

The bibliometric data extracted from WoSCC were then processed and analysed using a combination of manual curation and specialized bibliometric software tools. Microsoft Excel 2019 (Microsoft Corp, Redmond, WA, USA) was employed to organize descriptive statistics, such as the number of publications per year, citation trends, and author contributions. The BibTeX format was chosen for exporting bibliographic records, ensuring the inclusion of complete citation information and enhancing data integrity. Once the data were collated, they were imported into Biblioshiny, an R-based application of the Bibliometrix (https://www.bibliometrix.org/home/) package, which facilitates advanced bibliometric analysis and visualization.

The analysis involved several key bibliometric indicators to systematically examine research trends and impact in this field. Descriptive bibliometric indicators, including annual publication output, citation count distributions, and authorship metrics, were used to evaluate the growth and influence of research over time. A co-authorship analysis was conducted to identify collaborative networks among leading researchers and institutions contributing to the field. The journal impact analysis focused on identifying core journals that have consistently published high-impact research on AET for MSDs, considering metrics such as impact factors and H-indices.

To capture thematic trends and research focus shifts over time, keyword co-occurrence analysis was conducted to identify frequently occurring terms and evolving topics. This analysis provided insights into how the research focus has evolved, from initial investigations into the effects of aquatic exercise on specific musculoskeletal conditions to broader considerations of quality-of-life improvements and intervention standardization. The evolution of research themes was further examined through thematic mapping, highlighting the emergence of new topics and their relationships with established areas of study. In addition to keyword co-occurrence analysis, a citation network analysis was performed using VOSviewer (https://www.vosviewer.com/), a widely used bibliometric visualization tool that enables clustering of influential studies and identification of citation relationships between key publications. This approach allowed for the identification of foundational studies that have shaped the current understanding of AET in the management of MSDs. Throughout the analysis, efforts were made to ensure the robustness and reliability of the findings by cross-referencing results with previously published bibliometric studies in the field of rehabilitation and exercise science. The data analysis workflow was structured to minimize potential biases, such as those arising from database limitations or language restrictions, and to provide an accurate representation of the global research landscape.

It is important to acknowledge the limitations of the current study. The reliance on a single database, WoSCC, may not fully capture relevant studies indexed in other databases such as Scopus or PubMed, potentially leading to an incomplete representation of the field. Additionally, the exclusion of non-English articles may have introduced a language bias, limiting the generalizability of findings to predominantly English-language publications. Furthermore, although efforts were made to refine the search terms, the evolving terminology in the field may have resulted in the omission of certain relevant studies that used alternative descriptors for aquatic exercise interventions.

## Results

### Publication trend analysis

**Fig. 2:**
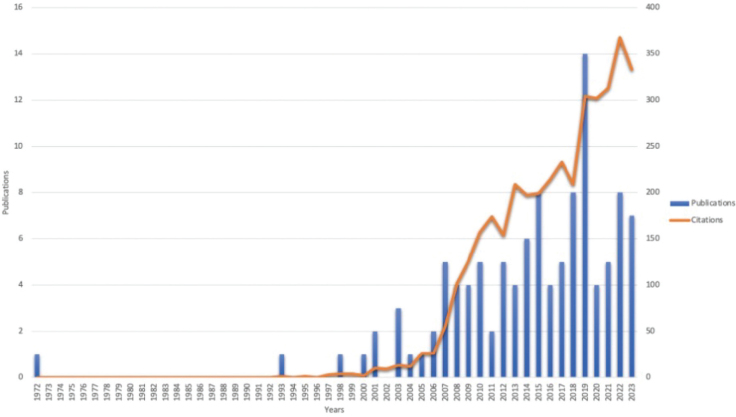
Chart of trends in publication delivery and citations.

Based on the selected search terms and inclusion criteria, we identified and analysed 117 articles published over a 52-year period from 1972 to 2024. These articles collectively received 4,070 citations, with an average of 34.79 citations per article and an H-index of 37. As shown in Fig. 2, the number of publications and citations in this field demonstrated a rapid growth trend from 2014 to 2024. During this 11-year period, 76 articles were published, accounting for 64.96% of the total publications. The peak in the number of published articles occurred in 2019 (*n* = 14), while the highest number of citations was recorded in 2022 (*n* = 349).

### Distribution of main authors

The distribution of leading authors is presented in Table I. The most prolific (*n* = 4) and influential (H-index = 4) author in the field of AET for MSDs is Mariana Arias Avila from the Department of Physiotherapy, Federal University of San Carlos, Brazil. The most cited author (*n* = 282) is Basia Belza from the School of Nursing, University of Washington, USA.

**Table I:** Top 4 main authors in aquatic exercise therapy research for managing musculoskeletal disorders

Rank	Authors	Countries	Document number	Citations	H-index
1	Avila, Mariana Arias	Brazil	4	41	4
2	Belza, Basia	USA	3	282	3
3	Donald Patrick	USA	3	149	3
4	Waller, Benjamin	Finland	3	166	3

### Country/region contribution

**Fig. 3:**
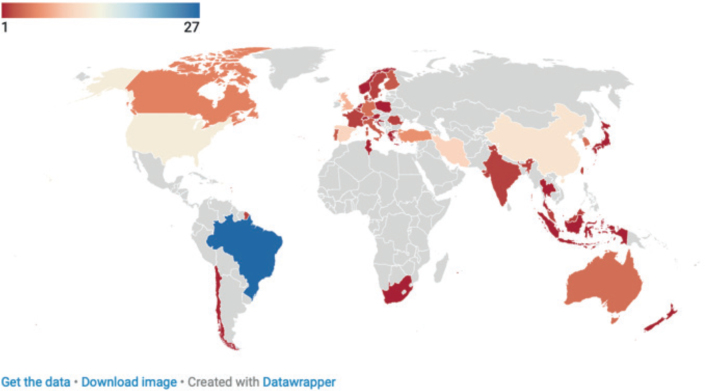
Geographic map of global research on aquatic exercise therapy as treatment for the management of musculoskeletal disorders.

We then assessed the current status of AET for MSDs studies in different countries. The literature was mainly provided by 39 countries/regions (Fig. 3). Brazil was the main contributor, with 27 publications (23.1% of the total), followed by the United States (13 publications, 11.1%) and China (12 publications, 10.3%). South America, especially Brazil, leads the field, while North America, Europe, and Asia also show significant contributions, albeit at a lower level. Spain, Turkey, and Finland were the main contributors in Europe, with other countries contributing fewer papers. Overall, research in this field has focused on South America and specific European and Asian countries, while other regions, including parts of Africa, have contributed less.

### Periodical distribution analysis

The research literature on AET for MSDs is published across 83 different scientific journals. Based on the criteria of publishing at least 3 articles, we present the top 8 journals in terms of publication volume in Table II. The most frequently published journal is *BMC Musculoskeletal Disorders* (*n* = 7), while the most influential journal, as indicated by its impact factor (CQ = 8.955), is *Arthritis & Rheumatism-Arthritis Care & Research*. Six of these 8 journals are ranked in the Q1 quartile.

**Table II:** Top 8 main journals publishing aquatic exercise therapy research for managing musculoskeletal disorders

Rank	Journal	Documents (% of 117)	JIF (2023)	CQ (2023)
1	BMC Musculoskeletal Disorders	7 (5.983)	2.2	Q2
2	Clinical Rehabilitation	4 (3.419)	2.6	Q1
3	American Journal of Physical Medicine Rehabilitation	4 (3.419)	2.2	Q1
4	Archives of Physical Medicine and Rehabilitation	4 (3.419)	3.6	Q1
5	Physical Therapy	4 (3.419)	3.5	Q1
6	Arthritis & Rheumatism-Arthritis Care & Research	3 (2.564)	8.955	Q1
7	Cochrane Database of Systematic Reviews	3 (2.564)	8.8	Q1
8	Rheumatology International	3 (2.564)	3.2	Q2

JIF: journal impact factor; CQ: category quartile.

### Most cited articles

Table III provided focuses on the 10 most cited articles concerning the effects of AET on osteoarthritis and fibromyalgia, of which “Does hydrotherapy improve strength and physical function in patients with osteoarthritis: a randomised controlled trial comparing a gym based and a hydrotherapy based strengthening programme” was the most cited, at 212 times (31). The second highest cited (*n* = 210) article is “Physical activity for osteoarthritis management: a randomized controlled clinical trial evaluating hydrotherapy or Tai Chi classes” (32). Both studies compare the measurable clinical benefits of water sports and land sports (gym sports and Tai Chi) for patients with osteoarthritis. In addition, 3 review articles (32–34) analysed the effects of AET on a series of results of different groups of people, including relieving pain, improving flexibility, and aerobic fitness. This is of great significance in synthesizing the evidence of AET and helps formulate clinical guidelines.

**Table III:** Top 10 cited articles

Rank	Title	Document type	Total citations	Journals
1	Does hydrotherapy improve strength and physical function in patients with osteoarthritis: a randomised controlled trial comparing a gym based and a hydrotherapy based strengthening programme (34)	Article	212	Annals of the Rheumatic Diseases
2	Physical activity for osteoarthritis management: a randomized controlled clinical trial evaluating hydrotherapy or Tai Chi classes (47)	Article	210	Arthritis and Rheumatism
3	Aquatic exercise for the treatment of knee and hip osteoarthritis (33)	Review	181	Cochrane Database of Systematic Reviews
4	Effects of aquatic exercise on flexibility, strength and aerobic fitness in adults with osteoarthritis of the hip or knee (48)	Article	148	Journal of Advanced Nursing
5	A randomized controlled trial of deep water running: clinical effectiveness of aquatic exercise to treat fibromyalgia (49)	Article	136	Arthritis and Rheumatism
6	Hydrotherapy versus conventional land-based exercise for the management of patients with osteoarthritis of the knee: a randomized clinical trial	Article	135	Physical Therapy
7	Aquatic exercise training for fibromyalgia (32)	Review	131	Cochrane Database of Systematic Reviews
8	Effectiveness of aquatic exercise for obese patients with knee osteoarthritis: a randomized controlled trial (50)	Article	97	PM R
9	Economic evaluation of aquatic exercise for persons with osteoarthritis (51)	Article	97	Medical Care
10	Systematic review and meta-analysis comparing land and aquatic exercise for people with hip or knee arthritis on function, mobility and other health outcomes (34)	Review	96	BMC Musculoskeletal Disorders

These 10 articles has been published in various sources, including high-impact journals such as *Rheumatology Yearbook*, *Arthritis & Rheumatism-Arthritis Care & Research*, and the *Cochrane Systematic Review Database*.

### Topic trend analysis

**Fig. 4:**
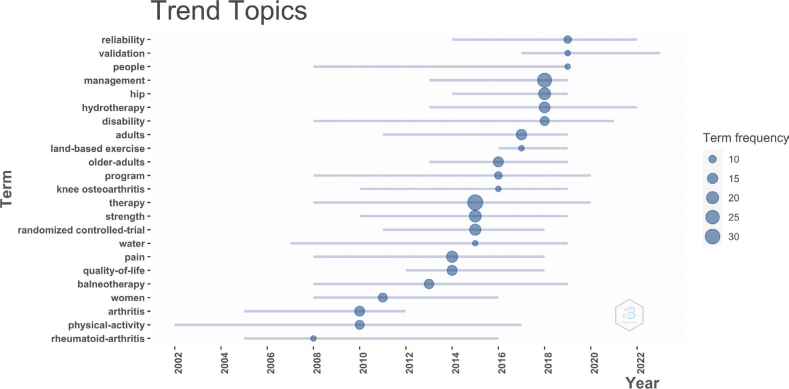
Trends in thematic focus in aquatic exercise therapy research for the management of musculoskeletal disorders.

Trend topic analysis is a crucial mapping tool that helps identify key themes and shifts in focus over time, indicating evolving research priorities. By checking keywords and maintaining a frequency of at least 9 words per year and a maximum of 7 words per year, different topics for different years were identified (Fig. 4). The details are as follows: 2008, “rheumatoid arthritis”; 2010, “physical activity and arthritis”; 2013, “balneotherapy”; 2014, “quality of life and pain”; 2015, “water, randomized control trial, strength and therapy”; 2016, “exercise, knee osteoarthritis, program and older adults”; 2017, “recommendations, land based exercise and adults”; 2018, “disability, hydrotherapy, and management”; 2019, “aquatic exercise, people, and the validation and reliability”. These thematic shifts indicate the broadening focus of AET research for MSDs, transitioning from disease-specific studies to a more holistic approach emphasizing functional rehabilitation and quality of life. This evolution highlights the growing importance of personalized, outcome-based interventions in the field.

## DISCUSSION

This bibliometric analysis offers valuable insights into the evolving trends and influence of AET for MSDs over the past 5 decades. The results indicate a significant growth in both the number of publications and citations, particularly from 2014 to 2024, highlighting the increasing interest in AET as a non-pharmacological approach to managing MSDs. This growth mirrors a broader shift in healthcare towards non-drug therapies, driven by a growing body of evidence supporting the benefits of AET for improving functional outcomes and quality of life in patients with MSDs.

### Publication growth and citation trends

The surge in publications and citations during the past decade reflects the growing recognition of AET’s efficacy in managing a variety of musculoskeletal conditions, such as osteoarthritis, fibromyalgia, and back pain. The peak in publication activity from 2014 to 2024 aligns with several global health trends, such as the ageing population and the increasing focus on chronic disease management through rehabilitation and exercise rather than medication alone. The rapid increase in citations, particularly in 2022, suggests that AET is gaining prominence not only in rehabilitation circles but also in mainstream clinical settings, where its potential to improve both physical function and pain management is increasingly recognized (23). This upward trajectory can also be attributed to the growing number of systematic reviews and meta-analyses that synthesize findings from individual studies, making the evidence more accessible and convincing to clinicians.

The significant rise in publications in 2019 and 2022 reflects important milestones in research funding and clinical adoption. It is likely that these increases were facilitated by policy shifts in healthcare that emphasize patient-centred care and cost-effective therapeutic alternatives, such as exercise-based treatments, particularly in the management of chronic, non-communicable diseases (23). This trend is in line with global healthcare initiatives that encourage non-invasive interventions for long-term disease management, especially given the economic burden of pharmacological treatments.

### Implications of country/regional contribution differentiation

The analysis of country/regional contributions shows that Brazil is the main contributor with 27 publications (23.1%), followed by the United States (*n* = 13, 11.1%) and China (*n* = 12, 10.3%). Brazil’s strong position may stem from its strong healthcare system focused on rehabilitation therapy and an academic environment that prioritizes physical therapy research. While contributions from the United States and China are significant, their research in AET is not as prominent, possibly because they are more focused on other forms of treatment. European countries such as Spain (10 papers, 8.5%) and Turkey (6 papers, 5.1%) are also active, reflecting the region’s interest in non-pharmacological treatments for MSDs. However, regions such as Africa and parts of Europe have fewer submissions, which may be due to limited research infrastructure, healthcare priorities, and economic factors. Despite these differences, global trends point to a growing interest in AET, indicating a growing recognition of AET as a viable treatment. More investment in research and infrastructure, especially in underrepresented regions, could further enhance global research outcomes in this field.

### Thematic shifts in AET research

One of the most striking findings from this analysis is the evolution of research themes over time. Early research primarily focused on specific conditions such as osteoarthritis and back pain, reflecting the need for targeted, disease-specific interventions. However, from 2014 onwards, there has been a clear shift towards broader outcomes, such as improving overall quality of life and functional mobility, and reducing disability. This shift is likely a response to the increasing recognition that MSDs not only impact physical health but also have significant psychosocial implications. The growing emphasis on holistic, patient-centred care, which addresses both physical function and psychological well-being, is evident in recent studies that incorporate multidimensional outcomes, including mental health and social participation. From the publication trend, this research field entered a stage of rapid development during 2013–2023, and the number of publications and citations increased significantly, especially the high number of publications since 2019 and the peak of citations in 2022, reflecting the continuous concern for this research topic in academic circles. This phenomenon may be due to the launch of the World Health Organization’s “Cooperation Plan for Bone and Joint Decade from 2000 to 2010” and its achievements (35, 36), which promoted people’s understanding of the burden of MSDs. With the obvious global population ageing trend, MSDs have become one of the main forms of disabling diseases of the elderly, leading to an increase in patients’ demand for non-drug and low-risk treatments. AET has been widely involved because of their low pressure on joints and good pain relief effect, and they have become established as one of the non-drug intervention therapies for the rehabilitation of MSDs.

The main author’s analysis shows the core strength of this field, but also reveals some problems. First, the authors who produce the most are not necessarily the most cited, indicating that there may be some imbalance between the number of studies and their influence. Second, the research in this field is mainly concentrated on a few authors and countries, which shows that the research on AET in the treatment of MSDs needs further internationalization and diversification to promote global sharing and promotion.

The periodical distribution analysis of AET research literature on MSDs reveals that the research in this field is mainly concentrated in a few high-impact periodicals, especially those covering rehabilitation medicine, physical therapy, and rheumatology. These journals are of high academic quality, reflecting the professional needs of the research topic and its academic value in specific fields and also providing a rich scientific basis for the practical application of this therapy in the management of MSDs. The most highly cited articles are published in highly influential journals, such as *Annals of the Rheumatological Diseases*, *Arthritis & Rheumatism-Arthritis Care & Research*, *Cochrane Database of Systematic Reviews*, etc. In addition, the higher impact factors and cited frequency of these journals further enhance the dissemination and influence of the research.

Several factors have contributed to this shift in focus. First, the increasing prevalence of MSDs in ageing populations has driven the need for rehabilitation protocols that promote long-term functional independence and mobility. As patients age, their needs extend beyond pain management to include the prevention of further disability and the maintenance of daily living activities. Second, there has been a growing recognition of the limitations of pharmacological treatments in providing sustainable relief, particularly for chronic conditions like osteoarthritis. This has further encouraged research into non-drug interventions such as AET that can address both the physical and psychological aspects of musculoskeletal diseases.

In order to further describe the development trend of AET for MSDs from 1972 to 2024, we divided the timeline into several stages based on the evolution of research themes. This division was determined by analysing shifts in thematic focus over time, which were identified through keyword co-occurrence analysis and citation patterns. By examining the frequency and relationships of keywords across different periods, we were able to pinpoint key transitions in research priorities, helping to categorize the literature into distinct stages that reflect the evolving focus of AET for MSDs.

*Early research and attention to the specific situation (before and in 2008).* The theme during this period is “rheumatoid arthritis”, which shows that early research mainly focuses on the therapeutic potential of AET for specific diseases such as rheumatoid arthritis. The research emphasizes the effectiveness of hydrotherapy in relieving joint inflammation and pain in patients with chronic MSDs, especially rheumatoid arthritis. Research shows that AET is safe and beneficial for controlling such symptoms (37).

*Expanding the scope of general medical benefits (2010–2013). The* theme in this period is “physical activity, arthritis, and bath therapy”. It shows that the research focus is extended to the broader health benefits of AET, including the impact on the management of arthritis as a physical activity. Bath therapy, as a water-based treatment method, also attracted attention during this period. Studies have found that this therapy can relieve pain and improve the mobility of patients with various types of arthritis (9, 38, 39).

*Emphasis on quality of life, pain management, and randomized trials (2014–2016).* The themes of this period are quality of life, pain, water, randomized controlled trials, strength, treatment, exercise, knee osteoarthritis, sports, and the elderly. It shows that the research began to emphasize the evaluation of the broader impact of AET on patients’ quality of life and pain relief. Randomized controlled trials began to dominate the literature, and the research focus shifted to specific diseases of specific populations, such as knee osteoarthritis in the elderly (40–42). These experiments help to establish that AET is a beneficial treatment for these people to keep healthy and relieve pain.

*Standardization and verification (2017–2023).* The theme in this period is “advice, land exercise, adults, disability, hydrotherapy, management, water sports, verification, and reliability”. At this stage, the development of AET research developed from solving specific diseases, such as rheumatoid arthritis, to broader health-related outcomes, such as quality of life and disability management. Researchers began to try to create a standardized AET plan, verify its effectiveness and ensure its reliability in managing disability (43, 44). In addition, the comparison with land movement also became a noteworthy research focus (45, 46).

Theme trend analysis reveals the evolution of AET in this field. This change shows that AET is gradually moving from clinical application to evidence-based medicine, trying to provide standardized and scientific solutions for actual health management.

### Future research directions

While significant progress has been made, several important research gaps remain. First, while AET has shown effectiveness in improving short-term outcomes, there is limited evidence on its long-term benefits. Future studies should focus on long-term follow-up of patients who undergo AET to determine its lasting impact on functional outcomes, disability prevention, and quality of life. Second, there is a need to explore how AET can be tailored for underrepresented populations, particularly those in low- and middle-income countries where access to aquatic facilities may be limited. Research into cost-effective models of delivery, such as community-based aquatic programmes, would help make AET more accessible and equitable. Third, integrating AET with emerging rehabilitation technologies, such as virtual reality and wearable sensors, holds great promise for enhancing the personalization and effectiveness of AET. These technologies can provide real-time feedback on patient performance, improve patient engagement, and allow for remote monitoring, making AET more adaptable and scalable. Combining AET with cognitive-behavioural therapy and strength training could further improve outcomes, especially for individuals with chronic pain or those recovering from surgery.

There are also some limitations to this study. First of all, the literature used comes from the Web of Science database, which may not cover all relevant high-quality research, especially the literature published in other databases, which may lead to limitation of the results. Second, we limited the language to English, which may lead to selection bias. Third, this study includes articles and comments, so there may be some deviations in reflecting the academic influence of articles through citation times.

### Conclusion

This study provides a comprehensive overview of the evolution of AET research for MSDs. The increasing focus on holistic, patient-centred outcomes reflects the growing importance of rehabilitation approaches that address both the physical and psychological aspects of musculoskeletal conditions. As the field progresses, future research must continue to explore innovative ways to integrate AET with emerging technologies, while also expanding its reach to underrepresented populations. By addressing these gaps, AET can play a key role in improving the lives of individuals with MSDs worldwide.
